# COVID-19: A national rise in penetrating trauma cared for by a prepared trauma system^☆^

**DOI:** 10.1016/j.sopen.2024.06.007

**Published:** 2024-07-03

**Authors:** Mallory Jebbia, Jeffry Nahmias, Matthew Dolich, Sebastian Schubl, Michael Lekawa, Lourdes Swentek, Areg Grigorian

**Affiliations:** University of California, Irvine, Department of Surgery, Division of Trauma, Burns and Surgical Critical Care, Orange, CA, USA

**Keywords:** COVID, Penetrating trauma, Firearms, Preparedness, Mortality

## Abstract

**Background:**

The COVID-19 pandemic negatively impacted the collective American psyche. Socioeconomic hardships including social isolation led to an increase in firearm sales. Previous regional studies demonstrated increased penetrating trauma during the pandemic but it is unclear if trauma systems were prepared for this influx of penetrating injuries. This study aimed to confirm this increased penetrating trauma trend nationally and hypothesized penetrating trauma patients treated during the pandemic had a higher risk of complications and death, compared to pre-pandemic patients.

**Methods:**

The 2017–2020 Trauma Quality Improvement Program database was divided into pre-pandemic (2017–2019) and pandemic years (2020). Bivariate analyses and a multivariable logistic regression analyses were performed controlling for age, comorbidities, injuries, and vitals on arrival.

**Results:**

From 3,525,132 patients, 936,890 (26.6 %) presented during the pandemic. The pandemic patients had a higher rate of stab-wounds (4.8 % vs. 4.5 %, *p* > 0.001) and gunshot wounds (5.8 % vs. 4.6 %, *p* < 0.001) compared to pre-pandemic patients. Among penetrating trauma patients, the rate and associated risk of in-hospital complications (5.0 % vs. 5.1 %, *p* = 0.38) (OR 0.98, CI 0.94–1.02, *p* = 0.26) was similar between pre-pandemic and pandemic cohorts but adjusted risk of mortality decreased during the pandemic (8.3 % vs. 8.3 %, *p* = 0.45) (OR 0.92, CI 0.89–0.96, *p* < 0.001).

**Conclusion:**

This national analysis confirms an increased rate of penetrating trauma during the COVID-19 pandemic, with a higher rate of gunshot injuries. However, this did not result in an increased risk of death or complications suggesting that trauma systems across the country were prepared to handle a dual pandemic of COVID and firearm violence.

## Introduction

### Background

The COVID-19 pandemic has had far-reaching consequences beyond its direct impact on public health, affecting various aspects of society and exacerbating existing socioeconomic and mental health issues [1]. In the United States, the pandemic has led to increased social isolation, job loss, and financial strain, contributing to a heightened sense of insecurity and anxiety among the population [[2], [3], [4], [5]]. This complex environment created fertile ground for increased firearm sales, as people sought various means to protect themselves and their families in uncertain times [6].

Previous research has proposed a connection between socioeconomic factors and psychological turmoil with an escalation in firearm-related violence [7]. Furthermore, higher degrees of social deprivation may correlate with increased firearm homicide rates [8,9]. The COVID-19 pandemic has amplified these underlying issues, leading to concerns about the potential implications for already stressed trauma systems nationwide [10].

Prior single center, regional and some multicenter studies have reported an increase in penetrating trauma, particularly gunshot wounds, during the pandemic [[11], [12], [13], [14]]. However, not all studies found an increase in penetrating trauma [13,14]. Thus, this study aimed to evaluate whether nationally there was an increase in penetrating trauma and if the pandemic has affected the outcomes of patients treated for penetrating trauma in overstretched trauma centers who were concomitantly dealing with the COVID-19 pandemic. We hypothesized an increased rate of penetrating trauma and that penetrating trauma patients treated during the pandemic had a higher risk of complications and death, compared to pre-pandemic penetrating trauma patients.

## Methods

The Trauma Quality Improvement Program (TQIP) database was utilized in this study and as a deidentified national database, the study was deemed exempt by our institutional review board and a waiver of consent granted. The 2017–2020 TQIP database was queried for adult patients 18 years and older. Patients were divided into pre-pandemic (2017–2019) and pandemic years (2020). This primary outcome was the rate of penetrating trauma in all patients before and after the COVID-19 pandemic began. We then subsequently performed a subset analysis of only penetrating trauma patients. Two groups were compared: penetrating trauma patients treated in the pre-pandemic years (2017–2019) and penetrating trauma patients treated in the pandemic year (2020). For this subset analysis the primary outcome was mortality and secondary outcome was development of any in-hospital complication including unplanned intubation, unplanned return to the operating room, pneumonia, acute respiratory distress syndrome (ARDS), organ space surgical site infection (SSI), superficial SSI, deep SSI, catheter associated urinary tract infection (CAUTI), central line associated blood stream infection (CLABSI), osteomyelitis, sepsis, cardiac arrest, cerebrovascular accident (CVA), deep venous thrombosis, pulmonary embolism, myocardial infarction, extremity compartment syndrome, pressure ulcer and acute kidney injury.

Demographic data points that were collected included age, sex, and comorbidities including congestive heart failure (CHF), chronic obstructive pulmonary disease (COPD), myocardial infarction (MI), cerebrovascular accident (CVA), peripheral arterial disease (PAD), chronic kidney disease, smoking, substance abuse and steroid use. The injury data collected included traumatic brain injury, thoracic injury, solid organ and hollow viscus injuries, as well as extremity and spine fractures. We also collected the injury severity score (ISS) and abbreviated injury scale (AIS) scores of the head/neck, face, chest, abdomen, spine, extremity and external regions. Vitals on arrival including heart rate (HR), respiratory rate (RR) and systolic blood pressure (SBP) were also recorded. Additional outcomes evaluated were intensive care unit (ICU) admission, ICU length of stay (LOS), and hospital LOS.

All analyses were performed with IBM SPSS Statistics for Windows (Version 29, IBM Corp., Armonk, NY). Bivariate analyses were first performed. A Mann-Whitney-*U* test was used to compare continuous variables and a chi-square was used to compare categorical variables in the bivariate analysis. Categorical data was presented as percentages while continuous data was presented as a median with interquartile range. We then performed a multivariable logistic regression analysis to determine the risk of mortality and risk of complications between the two time periods. We adjusted for potential confounders, which were selected based on discussion among coauthors, review of the literature and identifying univariate statistically significant differences between proposed confounding variables. These included age, ISS, hypotension on arrival, tachycardia on arrival, severe AIS (AIS >3) for the head, cirrhosis, COPD, baseline functional status (e.g., dependent or independent) and CKD [15,16]. *P*-values were defined as statistically significant if <0.05. The categorical variables included in our multivariable logistic regression analysis, such as yes/no metrics, had complete data. The only variables with some missing data were the continuous variables, heart rate and blood pressure, which collectively had missing data for only 1.2 % of patients. There was no missing data for patient age. Our logistic regression analysis was performed on cases with complete data for all variables.

To further evaluate the differences in penetrating traumas and outcomes between the pre-pandemic and pandemic periods, we performed a Difference-in-Differences (DiD) analysis using the General Linear Model (GLM) procedure. This approach allowed us to control for time-related factors and potential confounders [17]. We created an interaction term between the time period (pre-pandemic vs. pandemic) and the presence of penetrating trauma. We used Type III Sum of Squares to account for any unbalanced designs, and significance was set at an alpha level of 0.05. The parameter estimates provided coefficients for each predictor, allowing us to assess the main effects of time period and penetrating trauma, as well as the interaction effect on mortality and complications. This methodology enabled us to determine whether there was a significant difference in the mortality rates for penetrating trauma patients between the pre-pandemic and pandemic periods, while also accounting for potential confounding variables.

## Results

### Rates of penetrating trauma in the pre-pandemic versus pandemic cohorts

Of 3,525,132 patients, 936,890 (26.6 %) were treated during the pandemic. The pandemic patients had a higher rate of stab-wounds (4.8 % vs. 4.5 %, *p* > 0.001) and gunshot wounds (5.8 % vs. 4.6 %, *p* < 0.001) compared to pre-pandemic patients. Among the 352,624 penetrating trauma patients in the dataset, 248,325 (70.4 %) were pre-pandemic and 104,299 (29.6 %) were from the pandemic period.

### Demographics, comorbidities and vital signs for pre-pandemic vs pandemic penetrating trauma patients

Both pre-pandemic and pandemic penetrating groups were predominantly male with a median ISS of 5. The pandemic penetrating cohort had higher rates of smoking (31.7 % vs 31.1 %, *p* < 0.001) and substance abuse (12.7 % vs 11.4 %, p < 0.001) (Table 1). (See Table 2.)

**Table 1:** Demographics of pandemic and pre-pandemic penetrating trauma patients.

Characteristic	Pre-Pandemic Penetrating Patients	Pandemic Penetrating Patients	p-Value
	(*n* = 248,325)	(*n* = 104,299)	
Age, year, median (IQR)	33 (20)	32 (19)	<0.001
Male, n (%)	208,701 (84.1 %)	87,248 (83.7 %)	0.013
ISS, median (IQR)	5 (9)	5 (9)	<0.001
Penetrating mechanism, n (%)			
Stab wound	117,546 (47.3 %)	45,113 (43.3 %)	<0.001
Gunshot wound	116,741 (47.0 %)	53,636 (51.4 %)	<0.001
Other	14,038 (5.7 %)	5550 (5.3 %)	<0.001
Vitals on arrival, n (%)			
Hypotensive (SBP < 90)	22,396 (9.3 %)	9498 (9.4 %)	0.382
Tachypneic (RR > 22)	52,601 (22.2 %)	23,498 (23.5 %)	<0.001
Tachycardic (HR > 120)	30,183 (12.4 %)	13,129 (12.9 %)	<0.001
Comorbidities, n (%)			
Cerebrovascular accident	888 (0.4 %)	311 (0.3 %)	0.010
Diabetes	11,552 (4.7 %)	4707 (4.6 %)	0.320
Hypertension	28,666 (11.7 %)	11,902 (11.7 %)	0.818
Congestive heart failure	1337 (0.5 %)	570 (0.6 %)	0.571
Myocardial infarction	529 (0.2 %)	165 (0.2 %)	0.001
Anticoagulant Use	3065 (1.2 %)	1434 (1.4 %)	<0.001
Smoking	76,457 (31.1 %)	32,284 (31.7 %)	<0.001
Cirrhosis	970 (0.4 %)	288 (0.3 %)	<0.001
COPD	5002 (2.0 %)	1798 (1.8 %)	<0.001
Functional Dependence	1359 (0.6 %)	633 (0.2 %)	0.015
Substance abuse	28,074 (11.4 %)	12,912 (12.7 %)	<0.001
Chronic kidney disease	555 (0.2 %)	235 (0.2 %)	0.782

IQR = interquartile range, ISS = injury severity score, SBP = systolic blood pressure, RR = respiratory rate, HR = heart rate, COPD = chronic obstructive pulmonary disease.

**Table 2:** Injuries in pandemic and pre-pandemic penetrating trauma patients.

Injury n, (%)	Pre-Pandemic Penetrating Patients	Pandemic Penetrating Patients	p-Value
	(*n* = 248,325)	(*n* = 104,299)	
Brain	14,060 (5.7 %)	5702 (5.5 %)	0.022
Heart	4490 (1.8 %)	1761 (1.7 %)	0.014
Rib fracture	16,568 (6.7 %)	7386 (7.1 %)	<0.001
Lung	38,259 (15.4 %)	15,873 (15.2 %)	0.157
Diaphragm	7943 (3.2 %)	3305 (3.2 %)	0.645
Esophagus	382 (0.2 %)	137 (0.1 %)	0.112
Spleen	4478 (1.8 %)	1950 (1.9 %)	0.179
Liver	13,045 (5.3 %)	5618 (5.4 %)	0.107
Stomach	4919 (2.0 %)	2042 (2.0 %)	0.654
Small intestine	12,177 (4.9 %)	5392 (5.2 %)	<0.001
Colon	10,439 (4.2)	4706 (4.5 %)	<0.001
Rectum	1444 (0.6 %)	673 (0.6 %)	0.025
Kidney	5508 (2.2 %)	2387 (2.3 %)	0.196
Bladder	1459 (0.6 %)	681 (0.7 %)	0.022
Pelvic fracture	5845 (2.4 %)	2896 (2.8 %)	<0.001
Spine fracture	10,715 (4.3 %)	4800 (4.6 %)	<0.001
Cervical cord	1009 (0.4 %)	415 (0.4 %)	0.719
Spinal cord	3286 (1.3 %)	1557 (1.5 %)	<0.001
Upper extremity fracture	25,450 (10.2 %)	11,644 (11.2 %)	<0.001
Lower extremity fracture	22,721 (9.1 %)	11,409 (10.9 %)	<0.001

### Outcomes for pre-pandemic vs pandemic penetrating trauma patients

There was no difference in the rate of in-hospital complications between the two groups (pandemic 5.0 % vs. pre-pandemic 5.1 %, *p* = 0.38). The rate of mortality was similar in both groups (8.3 % vs 8.3 %, *p* = 0.45). There was no difference in median ICU LOS between the pre-pandemic or pandemic penetrating patients (3 vs 3 days, *p* = 0.37) (Table 3).

**Table 3:** Clinical outcomes in pandemic and pre-pandemic penetrating trauma patients.

Outcome	Pre-Pandemic Penetrating Patients	Pandemic Penetrating Patients	p-Value
	(n = 248,325)	(n = 104,299)	
LOS, days, median (IQR)	3.0 (4)	3.0 (3)	<0.001
ICU LOS, days, median (IQR)	3.0 (3)	3.0 (3)	0.374
Ventilator days, median (IQR)	2.0 (3)	2.0 (3)	0.091
Complications, n (%)	12, 693 (5.1 %)	5257 (5.0 %)	0.380
Cerebrovascular accident	316 (0.1 %)	129 (0.1 %)	0.782
Cardiac arrest	3512 (1.4 %)	1587 (1.5 %)	0.015
Myocardial infarction	143 (0.1 %)	48 (0.0 %)	0.177
Pneumonia/VAP	768 (0.3 %)	279 (0.3 %)	0.037
Acute respiratory distress syndrome	647 (0.3 %)	191 (0.2 %)	<0.001
Unplanned intubation	1219 (0.5 %)	519 (0.5 %)	0.800
Unplanned return to OR	2696 (1.2 %)	1290 (1.2 %)	0.181
Superficial surgical site infection (SSI)	529 (0.2 %)	202 (0.2 %)	0.247
Deep SSI	682 (0.3 %)	236 (0.2 %)	0.010
Organ space SSI	740 (0.3 %)	272 (0.3 %)	0.059
CAUTI	306 (0.1 %)	91 (0.1 %)	0.004
CLABSI	109 (0.0 %)	24 (0.0 %)	0.004
Sepsis	673 (0.3 %)	256 (0.2 %)	0.175
Acute Kidney injury	1298 (0.5 %)	551 (0.5 %)	0.839
Deep vein thrombosis	1596 (0.6 %)	634 (0.6 %)	0.231
Embolism	721 (0.3 %)	333 (0.3 %)	0.152
Extremity compartment syndrome	432 (0.2 %)	212 (0.2 %)	0.064
Mortality, n (%)	20,498 (8.3 %)	8690 (8.3 %)	0.447

LOS = length of stay, IQR = interquartile range, ICU = intensive care unit, VAP = ventilator-associated pneumonia, OR = operating room, CAUTI = catheter-associated urinary tract infection, CLABSI = central line-associated bloodstream infection.

After controlling for potential confounders, the associated risk of in-hospital complications (OR 0.98, CI 0.94–1.02, *p* = 0.26) was similar between the pre-pandemic and pandemic cohorts (Table 4). However, after adjustment, the associated risk of mortality decreased during the pandemic (OR 0.92, CI 0.89–0.96, *p* < 0.001) when compared to the pre-pandemic cohort. The greatest predictor of mortality was hypotension on admission (OR 32.04, CI 30.68–33.46, p < 0.001) (Table 5).

**Table 4:** Multivariable logistic regression analysis for risk of complications for pre- versus post-pandemic penetrating trauma patients.

Risk factor	OR	CI	p-Value
Pandemic vs pre-pandemic	0.98	0.94–1.02	0.259
Age (years)	1.01	1.00–1.01	<0.001
Injury severity score ≥ 25	7.44	7.13–7.76	<0.001
Vitals on arrival			
Hypotension	1.84	1.76–1.92	<0.001
Tachycardia	2.10	2.02–2.19	<0.001
Severe head injury (AIS > 3)	0.70	0.66–0.74	<0.001
Comorbidities			
Cirrhosis	2.25	1.84–2.74	<0.001
COPD	1.33	1.19–1.48	<0.001
Functional deficit	1.86	1.57–2.20	<0.001
Chronic kidney disease	2.03	1.57–2.62	<0.001

AIS = abbreviated injury scale, COPD = chronic obstructive pulmonary disease.

**Table 5:** Multivariable logistic regression analysis for risk of mortality for pandemic versus pre-pandemic penetrating trauma patients.

Risk factor	OR	CI	p-value
Pandemic vs pre-pandemic	0.92	0.89–0.96	<0.001
Age (years)	1.01	1.01–1.01	<0.001
Injury severity score ≥ 25	9.53	9.12–9.96	<0.001
Vitals on arrival			
Hypotension	32.04	30.68–33.46	<0.001
Tachycardia	0.92	0.88–0.97	0.002
Severe head injury (AIS > 3)	20.40	19.30–21.57	<0.001
Comorbidities			
Cirrhosis	1.82	1.40–2.36	<0.001
COPD	0.87	0.75–1.01	0.059
Functional deficit	1.08	0.85–1.37	0.521
Chronic kidney disease	1.72	1.24–2.40	<0.001

AIS = abbreviated injury scale, COPD = chronic obstructive pulmonary disease.

After performing a DiD analysis regarding complications, the interaction between the pre-pandemic and pandemic periods and penetrating traumas was not significant (F = 0.006, *p* = 0.939), suggesting no significant difference in the effect of the pandemic period on complication rates for penetrating trauma patients. In contrast, for mortality, the interaction between the pre-pandemic and pandemic periods and penetrating traumas was significant (F = 5.130, *p* = 0.024), indicating a significant difference in the effect of the pandemic period on mortality rates for penetrating trauma patients.

## Discussion

Penetrating trauma continues to be a significant scourge of American Society, with rates that are almost four times more than other industrialized nations [18]. In this study, we analyzed the impact of the COVID-19 pandemic on the incidence of penetrating trauma and their outcomes across the United States. Similar to some smaller regional studies, there was a national increase in the rate of both stab wounds and gunshots, with the latter having an over 25 % relative increase in the rate of gunshot violence during the pandemic compared to the pre-pandemic period. However, despite this increase, there was no difference in the associated risk of complications when comparing the pre-pandemic to pandemic cohort. Interestingly, the associated risk of mortality in penetrating trauma patients was actually slightly lower during the pandemic compared to the pre-pandemic period, suggesting that overall, trauma systems nationally were prepared to handle the dual challenges of COVID-19 and increased penetrating trauma.

The pandemic influenced the landscape of trauma surgery. Klutts et al. found in their county-wide study there was a significant rise in penetrating traumas during the pandemic [11]. Yeates et al. similarly found a rise in the rate of penetrating trauma across southern California after stay-at-home orders went into effect [12]. The social distancing and stay-at-home orders during the pandemic may have decreased overall emergency room visits in the early weeks of the pandemic, however, Pelzl et al. found these social distancing orders led to a sharp rise in the rate of penetrating trauma accompanied by a rise in injury severity during that period [19]. This national analysis confirms these findings with an increase in both stab wounds and a more pronounced increase in the rate of firearm violence. Some speculate that stay-at-home orders led to closure of support groups and community organizations that are instrumental in preventing firearm violence and therefore contributed to the rise in firearm-related injuries during the pandemic [20]. It is essential to understand the complex interplay of factors that contributed to this increased incidence of penetrating trauma, which may include increased domestic violence, gang activity, or civil unrest during the pandemic [21]. Harmon-Darrow et al. found that restorative justice interventions and mediation programs did significantly reduce rearrest and reincarceration rates [22]. The increase in firearm sales during the pandemic may have played into the trend seen nationally and highlights the need for continued monitoring and public health interventions, such as mediation programs, to mitigate the ongoing and increased risk of firearm violence during a pandemic or other eras of crisis.

The influx of penetrating trauma during the pandemic, coupled with the large overflow of COVID-19 patients may have had an impact on clinical outcomes. Interestingly, this study found that despite the increased incidence of penetrating trauma, there was no significant rise in complications among penetrating trauma patients treated during the pandemic. We additionally found no significant difference in total hospital LOS between the pandemic and pre-pandemic groups. This is congruent with the regional findings of Klutts et al. reporting a rise in penetrating trauma during the pandemic which was not associated with worse outcomes [11]. However, in contrast, another single-center study found a rise in penetrating trauma during the pandemic which was associated with increased ICU LOS and ventilator days. However, the difference in findings may be explained by the fact that their pandemic group had a significantly higher proportion of pre-existing conditions leading to differences in hospital complications and outcomes [10]. Alternatively, the overall decrease in trauma patients due to less blunt trauma during the pandemic may have allowed for a less overwhelmed trauma service and therefore lower complication rate despite increased penetrating trauma. Our study is the first to evaluate the outcomes of penetrating trauma on a national level, and it shows that despite the rise in penetrating trauma, our nationwide trauma system was prepared and treated patients in a way that maintained quality of care. This may be attributed to the adaptability and resilience of trauma systems across the country, which managed to maintain the quality of care under challenging circumstances. The swift implementation of telemedicine, reorganization of trauma teams, and increased attention to infection prevention may have played a crucial role during this difficult time period. This is particularly important for future nation- and world-wide events that can result in increased trauma rates, as it shows proper planning can prevent a rise in morbidity.

The pandemic may have had an impact on the risk of mortality for trauma patients treated during the pandemic [23]. Interestingly, our data suggests that the risk of mortality for penetrating trauma actually improved during the pandemic. Several factors may explain this finding. It is possible that advances in trauma management and other adaptations made during the pandemic have contributed to this improvement. Or that because elective surgeries were cancelled in many hospitals, operating rooms were more readily available. Alternatively, there may be other confounders not accounted for and in fact the risk of mortality may just be similar across time periods which still would be an important finding given the context of increased penetrating trauma. Meaning that national trauma centers and our existing trauma systems were prepared to manage this double pandemic of COVID and firearm violence. Regardless, future research is needed to identify factors that contributed to the success of these systems, which could inform further efforts to strengthen trauma system infrastructure and preparedness.

Limitations of this study include those associated with database studies. First, it is retrospective, so it is subject to selection bias and missing or misclassified data. Second, as a nationwide study, it aggregates data from a variety of healthcare settings across the country, each with its own unique circumstances and responses to the pandemic. Therefore, the trends observed may not reflect the experience at every individual institution. Additionally, the timing and implementation of stay-at-home orders varied across the country and the study period only covers the initial year of the pandemic. Furthermore, the database lacks pertinent risk factors for firearm violence such as social determinants of health [7] and granular data regarding the circumstances surrounding the gunshot and stab wounds included. Finally, the database is confined to index hospitalization and thus lacks long-term data and patient-centric outcomes.

## Conclusion

This national analysis spanning four years of data confirmed a national increase in the rate of penetrating trauma, particularly gunshot wounds, across the United States during the initial period of the COVID-19 pandemic. However, this did not result in an increased risk of complications or mortality, suggesting that trauma systems nationwide were well prepared to handle the dual pandemic of COVID-19 and firearm violence. These findings underscore the importance of continued investment in trauma infrastructure and preparedness to effectively respond to future public health crises. In addition, further investment is needed in resources and primary prevention efforts to curtail firearm violence in America.

This project was reviewed by our institutional review board and deemed exempt, a waiver of consent was granted (see Methods section).

## CRediT authorship contribution statement

**Mallory Jebbia:** Writing – original draft, Conceptualization. **Jeffry Nahmias:** Writing – review & editing, Formal analysis. **Mathew Dolich:** Writing – review & editing. **Sebastian Schubl:** Writing – review & editing. **Michael Lekawa:** Writing – review & editing, Data curation. **Lourdes Swentek:** Writing – review & editing. **Areg Grigorian:** Writing – review & editing, Supervision, Formal analysis, Conceptualization.

## Declaration of competing interest

We have no conflicts of interest or financial disclosures.
